# Evaluation of Organically Versus Synthetically Derived Multivitamin and Mineral Supplementation on Circulatory Concentration in Healthy Humans: A Single-Blinded Randomized Intervention Study

**DOI:** 10.1016/j.cdnut.2026.107676

**Published:** 2026-03-16

**Authors:** Rachel Churm, Eimear Sutton, Jason P Pitt, Richard M Bracken

**Affiliations:** 1 Applied Sports Technology Exercise and Medicine Research Centre (A-STEM), Faculty of Science and Engineering, Swansea University, Swansea, United Kingdom; 2 Diabetes Research Group, Swansea University, Swansea, United Kingdom; 3 BIOVIT Ltd., Tenby, United Kingdom

**Keywords:** multivitamin supplementation, nutrition, organically sourced, synthetic sourced, supplementation

## Abstract

**Background:**

Growing consumer interest in organic products has stimulated demand for organically sourced multivitamins and minerals (MVM); however, evidence comparing their efficacy with conventional synthetic formulations is limited.

**Objectives:**

This study aims to assess circulatory concentrations of organically sourced (ORG) compared with synthetically produced (SYN) MVM supplements.

**Methods:**

Single-blind randomized 30-d intervention, in which healthy adults (*n* = 62) ingested either ORG or SYN-sourced MVM formulations. Circulating MVM concentrations were assessed at baseline and postintervention. A subset of participants (*n* = 19) completed an acute crossover trial for assessment 2 h postingestion.

**Results:**

Both ORG and SYN produced comparable changes in circulating micronutrient concentrations across 2-h and 30-d ingestion protocols, with no significant changes between groups for all vitamins and minerals. Over 30-d ingestion resulted in significant within-group changes; elevated biotin concentrations over time (*F* = 5.36, *P* = 0.022), and B9 folate (*F* = 5.46, *P* = 0.021), in both the ORG (*P* < 0.05) and SYN groups (*P* < 0.05). No changes were observed for vitamin B12, vitamin C, vitamin D, calcium, zinc, iron, ferritin, and transferrin concentrations. Two-hour ingestion resulted in significant increases in vitamin B12, vitamin D, calcium, zinc, iron, and ferritin for both supplement types; however, elevated folate and transferrin were present in ORG only (both *P* < 0.01).

**Conclusion:**

Organic and synthetic MVM formulations demonstrated similar postingestion circulatory profiles. These findings support the use of organic MVMs as functionally comparable alternatives to SYN supplements, aligning with increasing consumer demand for organic, naturally derived health products.

## Introduction

Micronutrients such as vitamins and minerals are essential for maintaining health and preventing disease [1]. Micronutrient deficiencies represent a major global public health challenge [2,3], yet the true burden of mortality and morbidity remains uncertain [4]. These deficiencies can arise from inadequate intake of 1 or more essential vitamins or minerals because of restricted caloric intake, whether intentional for weight management or unintentional due to food insecurity, illness, or aging [4]. Importantly, deficiencies may also occur in individuals consuming sufficient or excessive calories when dietary patterns exclude key food groups or limit micronutrient-dense foods. Fortification and multivitamin and mineral (MVM) supplementation; therefore, represent effective public health strategies to reduce such deficiencies worldwide [5].

Developing vitamins for fortification and MVM supplementation from organic sources, such as plant extracts, fermented microorganisms, or algae grown under certified organic conditions, offers potential economic, ethical, and physiological advantages. The global organic food sector has expanded substantially over the past 2 decades and is projected to reach US $361.7 billion by 2030 [6,7]. Growth is driven by increasing public awareness of the health, environmental, and ethical implications of food production [8,9]. Emerging evidence indicates that organically grown produce tends to contain lower concentrations of pesticide residues, nitrates, and heavy metals, and in some cases exhibits higher concentrations of antioxidant compounds [10,11]. However, these differences are generally within established regulatory safety limits, and their direct clinical relevance remains uncertain.

An emerging body of literature suggests the potential that organic production may enhance nutrient bioefficacy through synergistic phytochemical interactions and improved bioavailability [12,13]. Nonetheless, this uncertainty regarding their bioavailability and efficacy compared with synthetic equivalents presents as a significant barrier to industry adoption of organic vitamins and minerals. In addition, scaling the production of organic vitamins remains challenging because of higher production costs, limited raw material availability, and technological barriers in extraction and stabilization [14,15]. As a result, most commercial fortification and MVM products rely on synthetic vitamins produced through laboratory processes and minerals from mined rocks, rather than nutrients sourced from organic foods. Although organically derived micronutrients originate from plant, algal, or microbial sources, their production nevertheless involves controlled extraction, filtration, and quality-assurance procedures conducted in accordance with established manufacturing standards. Synthetic vitamins and minerals are synthesized from nonfood starting materials and produced under regulated conditions designed to ensure safety, stability, and consistency [1,16]. The principal distinction; therefore, lies in the origin and processing pathway of the nutrient sources, rather than in the absence of industrial manufacturing.

The biochemical source of micronutrients, whether synthetic, semisynthetic, or plant/microbe-derived, may influence bioavailability and physiological utilization. Although synthetically sourced vitamins and minerals are chemically similar to their natural counterparts, they lack the broader phytochemical matrix present in whole-food sources [17]. Emerging evidence suggests that synthetic vitamins, whereas generally effective in correcting deficiencies, may not fully replicate the metabolic or functional outcomes achieved with naturally derived compounds [16]. Despite increasing consumer demand for organically aligned products [8], few systematic human studies have directly compared the bioavailability, metabolism, and physiological effects of organic with synthetic vitamins.

The present study aims to characterize changes in circulating vitamin and mineral concentrations after ingestion of 60% of the recommended daily allowance (RDA) of either organically or synthetically derived MVM supplements in healthy adults. This controlled comparison will enable evaluation of differences in bioavailability, retention, and short-term physiological responses, helping bridge the gap between consumer perception and measurable biochemical outcomes.

## Methods

### Participants

This study was conducted according to the Declaration of Helsinki, and all procedures involving human subjects were approved by the Faculty of Science and Engineering, Swansea University Research Ethics Committee; approval number: 2 2024 9146 824764. Both male and female participants [age: 33.0 ± 12.8 y, estimated body fat: 24 ± 8.7%, BMI (in kg/m^2^): 25.0 ± 3.6] were recruited to this study. 62 participants were enrolled in the study. Participants were excluded if they had known or suspected intolerance to products, suffered from a history of life-threatening disease, had any chronic health condition, had surgery or trauma with significant blood loss (>500 mL) within the last 3 mo prior to screening, had a systolic blood pressure measurement of <90 mmHg during screening and/or currently pregnant or planning on becoming pregnant during the study period. Written informed consent was obtained from all participants.

### Experimental design

**FIGURE 1:**
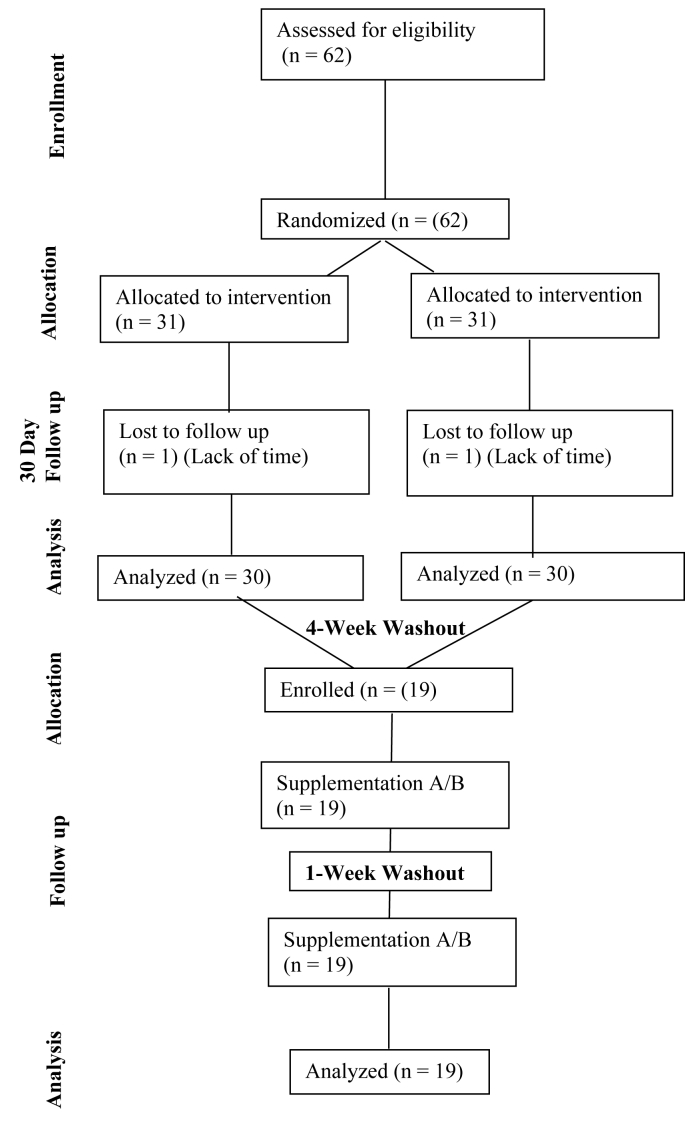
CONSORT diagram showing the flow of participants through each stage of a randomized trial.

In a randomized, single-blind intervention design study, participants (*n* = 62) were allocated to complete a 30-d, daily consumption of either an organic (ORG, BIOVIT Blend, Biovit) or synthetic (SYN, PREMIX Blend, Prinova Solutions) supplement (Figure 1). The principal investigator was responsible for generating the random allocation sequence (via National Cancer Institute Clinical Trial Randomization Tool), enrolling participants, and assigning interventions. There were no reported losses and exclusions after randomization in any group. Because of the nonclinical nature of the randomized study, it was not registered at inception.

### Supplementation

Participants were randomly assigned to either the ORG or SYN trial. After an overnight fast, participants arrived at the laboratory and were observed consuming 10 g maltodextrin powder containing either synthetic or organically sourced MVM mix (from a commercially available brand; BIOVIT Ltd., manufactured by BIOVIT). Both ORG and SYN sachets were produced in a blinded format from Reading Scientific Services Ltd. (https://www.rssl.com/), both ORG and SYN sachets contained maltodextrin powder, and were matched for weight (10 g). Appearance, including consistency and color, was well matched across groups. Compositional breakdown was equivalent to 60% RDA of each mineral or vitamin component as listed in Table 1. Certificates of analysis from manufacturers provided verification of the amount of the individual nutrient to ensure compliance; ingredient tolerances are set at ±20% of the specified level. Ten grams sachet of organic maltodextrin and either ORG or SYN MVM mix were given to participants in blinded sachets, 1 sachet was given to participants for consumption of 1 serving (containing 60% RDA) on their first experimental trial visit. Participants were provided with 30 d of sachets, with participants consuming 1 each day prior to returning to the laboratory within 24–48 h of finishing the vitamin schedule. Participants were instructed to mix the sachet with a glass of water and consume it straight away.

**TABLE 1:** Mineral and vitamin list for both ORG and SYN supplement, developed to 60% RDA for each ingredient.

Nutrient	Source (ORG complex)	Natural active components (ORG complex)	Source (SYN complex)	Nutrient concentration (mg) (eq. 60% RDA)
Vitamin B12	Organic shiitake mushroom extract	Mix of cobalamins: Methylcobalamin, Hydroxocobalamin, Adenosylcobalamin	Cyanocobalamin 0.1% on maltodextrin	0.0015
Vitamin D3	Organic lichen extract	Cholecalciferol	Cholecalciferol	0.003
Biotin	Organic holy basil extract, organic sunflower seed extract	D-biotin	D-biotin 1% on maltodextrin	0.03
Folate	Organic spinach extract	Tetrahydrofolate (and its derivatives, including L-Methylfolate (5-MTHF))	Folic acid 10% on maltodextrin	0.12
Selenium	Organic holy basil extract, organic sunflower seed extract	Proteinate forms (Selenomethionine) and methylated forms (Se-methylselenocysteine)	Sodium selenite 1% on maltodextrin	0.033
Vitamin C	Organic amla extract	L-ascorbic acid	Ascorbic acid	48
Chromium	Organic curry leaf extract and organic parsley extract	Organic acid forms (Chromium citrate, Chromium malate, Chromium acetate)	Chromium chloride 6H_2_O (5% Cr) SD on maltodextrin	0.024
Calcium	Organic red algae extract	Calcium carbonate	Calcium carbonate	480
Iodine	Organic hebridean seaweed extract	Iodide	Potassium iodide 5% on maltodextrin	0.09
Iron	Organic curry leaf extract	Nonheme iron [e.g., Iron (III) citrate]	Ferrous sulfate, dried	8.4
Zinc	Organic guava leaf extract	Zinc lysinate, glycinate, aspartate, malate, ascorbate, stearate, palmitate	Zinc citrate 2H_2_O	6

Abbreviations: ORG, organically sourced; RDA, recommended daily allowance; SYN, synthetically produced.

### Subset acute administration study design

A subset of 19 individuals participated in a blinded crossover trial, following a 4-wk washout period after the main trial, providing a baseline and 2-h (at rest) postingestion blood sample after consuming either ORG or SYN supplement, as detailed above. Both male and female participants (aged: 33.2 ± 10.6 y, estimated body fat: 26 ± 8.3 %, BMI: 26 ± 4.4) were recruited to this substudy. All participants carried out a crossover design, ingesting both ORG and SYN supplements on separate days, with at least a 1-wk washout period between testing.

### Experimental protocol

Prior to participation in both the experimental trials, participants were familiarized with the laboratory equipment and the test procedures. On the morning of the test, participants reported to the Exercise Physiology Laboratory after a >10 h fast and 24-h abstinence from strenuous exercise, where measures of body mass (weighing scales; Seca 770 Digital Scales, Seca Ltd.), height (stadiometer; Holtain Stadiometer, Holtain Ltd.), and estimated fat percentage using bioelectrical impedance analysis (Bodystat Quadscan 4000, Bodystat Ltd.) were made while wearing minimal clothing. Participants were then seated for a 10-min period while venous blood samples were collected into dipotassium ethylenediaminetetraacetic acid (EDTA-K2) BD Vacutainer tubes and BD Vacutainer serum-seperating tube (SST) II Advance Tubes.

For the acute trial, participants were required to ingest the blinded supplement and remain within the laboratory with minimal exertion for 2 h, during which a secondary blood sample was collected.

### Diet control

Participants were strongly encouraged and reminded to stick to their habitual diets during trial periods. Participants were also instructed not to perform any physical activity in the 24 h period prior to each trial.

### Blood analyses

All venous blood samples were treated immediately and centrifuged (Heraeus Megafuge 8, Thermo Scientific) for plasma 10 min at 2000 × *g* and for sera, 10 min at 1500 × *g* after 10 min of clotting incubation. Extracted plasma and sera were frozen immediately (−80°C) for later analysis of mineral and vitamin concentrations using either a fully-automated clinical chemistry analyzer (RX DAYTONA+; Randox and Forth Ltd.; Forth) or commercially available enzyme-linked absorbent assays (ELISA, ABBEXA). RX DAYTONA+ (Randox) assessed sera concentrations of, with lower limit of detection (LLOD) provided; iron (μmol/L, LLOD: 1.89 μmol/L), calcium (mmol/L, LLOD: 2.12 mmol/L), ferritin (ng/mL, LLOD: 5.9 ng/mL), transferrin (mg/dL, LLOD: 187 mg/dL), and zinc (μmol/L, LLOD: 2.4 μmol/L). Plasma concentrations of folate (μg/L; LLOD: 1.4 μg/L), vitamin B12 (pmol/L; LLOD: 27 pmol/L), and vitamin D (nmol/L; LLOD: 11 nmol/L) were analyzed via an external laboratory, Forth Ltd. (Forth). Commercially available ELISAs (ABBEXA) were used to assess serum biotin (pg/mL) and vitamin C (μg/mL) concentrations. The LLOD for the assay was 13 pg/mL and 0.18 μg/mL, respectively. Average intra-assay coefficients of variation were 6.0% and 7.2%, respectively. Data that surpassed the limits of detection were excluded from analysis.

### Data analysis

The data were analyzed using the Statistical Package for the Social Sciences software (version 26, SPSS Inc.). Data were reported as means ± SD, with *P* < 0.05 accepted. All data were assessed for normality (Shapiro–Wilk’s test), all variables were deemed to be normally distributed and subsequently analyzed using 2-way repeated measures analysis of variances (condition × time) with post hoc dependent *t*-tests conducted with Bonferroni corrections where appropriate. Paired samples *t*-test was used to compare the within-intervention time effect. This pilot study could not generate a priori power on primary endpoints due to a lack of published information.

## Results

Baseline characteristics for both groups were well matched for sex, age, mass, BMI, waist circumference, and body fat mass (%). In addition, there was no significant difference in baseline circulatory concentrations of vitamins and minerals (Table 2).

**TABLE 2:** Baseline characteristics of synthetic and organic trial groups.

	Synthetic (*n* = 30)	Organic (*n* = 30)	*P*
Male [% (n)]	17	17	
Age (y)	34 ± 14	32 ± 12	0.47
Mass (kg)	74 ± 16	71 ± 12	0.43
BMI (kg/m^2^)	25 ± 4	24 ± 3	0.22
Waist (cm)	80 ± 10	77 ± 9	0.31
Fat mass (%)	25 ± 9	22 ± 8	0.35
Mineral and vitamin concentration day 0			
Vitamin B12 (pmol/L)	94 ± 44	81 ± 28	0.17
Folate (μg/L)	5.1 ± 4.0	6.2 ± 3.7	0.33
Vitamin D (nmol/L)	62 ± 32	65 ± 38	0.80
Iron (μmol/L)	16.9 ± 8	17 ± 5.5	0.93
Calcium (mmol/L)	2.43 ± 0.17	2.2 ± 0.12	0.89
Ferritin (ng/mL)	130 ± 106	103 ± 63	0.24
Transferrin (mg/dL)	266 ± 33	265 ± 34	0.91
Zinc (μmol/L)	7.9 ± 2.7	8.3 ± 2.6	0.61
Biotin (nmol/L)	1.3 ± 0.9	2 ± 1	0.31
Vitamin C (μg/mL)	48 ± 44	41 ± 22	0.42

### Chronic supplementation of organic versus synthetic supplementation

#### Bioavailability of vitamins and minerals

Resting vitamin and mineral concentrations were similar between condition groups at baseline (Table 2).

**FIGURE 2:**
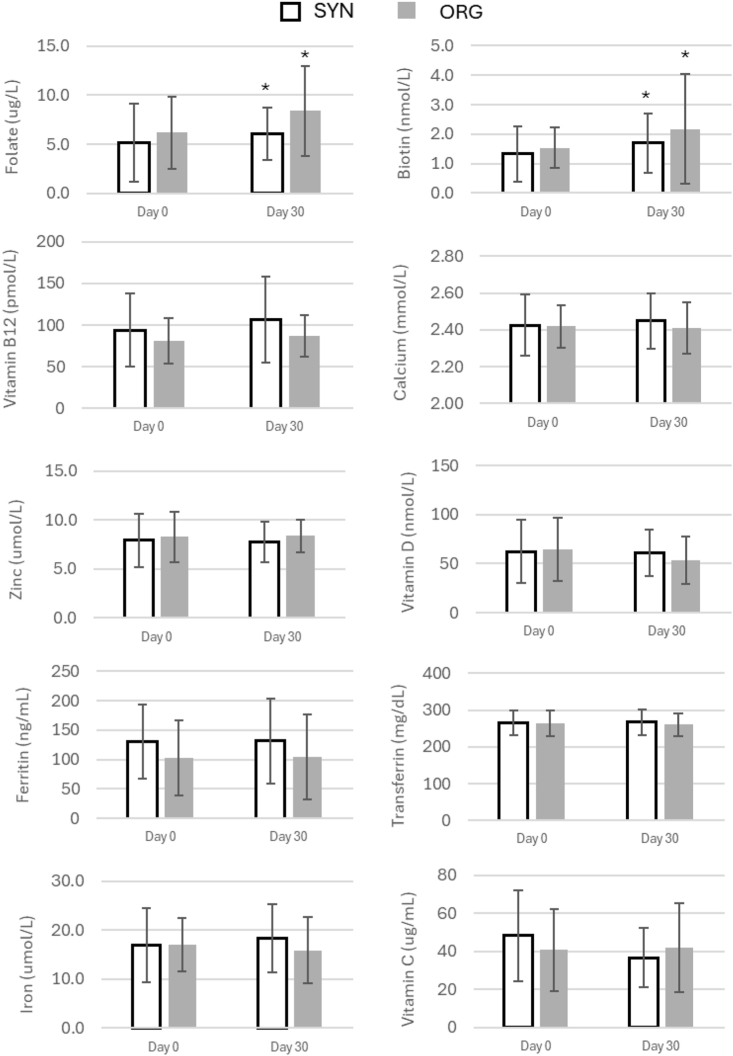
Vitamin and mineral circulatory concentrations for synthetic sourced MVM supplementation (black outline) and organic sourced MVM supplementation (gray solid) trials at rest (day 0) and at post 30-d ingestion (day 30). (∗) Indicates significant difference across time compared with rest analyzed using paired sample *t*-test (*P* ≤ 0.05). Data reported as mean ± SD. MVM, multivitamins and minerals; ORG, organically sourced; SYN, synthetically produced.

Biotin (vitamin B7): There was a significant main effect of time on circulatory biotin concentrations (*F* = 5.36, *P* = 0.022). Mean concentrations increased from day 0 (1.432 nmol/L) to day 30 (1.938 nmol/L), indicating an overall rise over the 30-d period. Post 30-d ingestion concentrations were significantly elevated across time for B7 biotin in both the ORG (day 0 compared with day 30; 1.54 ± 0.7 compared with. 2.17 ± 1.9 nmol L^−1^, *P* < 0.05) and SYN groups (day 0 compared with day 30 post; 1.32 ± 0.9 compared with 1.69 ± 1.0 nmol L^-1^, *P* < 0.05) (Figure 2).

No significant main effect of treatment was observed (*F* = 2.42, *P* = 0.122), with comparable values in the ORG (1.856 nmol/L) and SYN (1.515 nmol/L) groups. The time × treatment interaction was not significant (*F* = 0.34, *P* = 0.560), demonstrating that the increase in biotin concentrations over time occurred similarly across treatments.

Folate (vitamin B9): a significant main effect of time was observed for B9 folate (*F* = 5.46, *P* = 0.021), with concentrations increasing from day 0 (5.73 μg/L) to day 30 (7.23 μg/L). Post 30-d ingestion concentrations were significantly elevated in both the ORG (day 0 compared with day 30; 6.2 ± 3.7 compared with 8.4 ± 4.6 μg/L) and SYN groups (day 0 compared with day 30 post; 5.1 ± 4.0 compared with 6.1 ± 2.7 μg/L, *P* < 0.01) (Figure 2).

A significant treatment effect was also detected (*F* = 6.48, *P* = 0.012), with higher concentrations in the ORG group (7.29 μg/L) compared with the SYN group (5.66 μg/L), irrespective of time point. No significant interaction effect was present (*F* = 1.16, *P* = 0.283), indicating that both groups changed in a similar manner over time, but maintained a clear difference in overall folate status.

Vitamin B12: vitamin B12 showed no significant main effect of time (*F* = 1.69, *P* = 0.196), with similar values at day 0 (87.91 pmol/L) and day 30 (97.10 pmol/L). However, a significant treatment effect was detected (*F* = 5.87, *P* = 0.017), with the SYN group exhibiting higher mean values (101.07 pmol/L) than the ORG group (83.94 pmol/L). There was no significant interaction (*F* = 0.18, *P* = 0.671), highlighting that the SYN group had consistently higher vitamin B12 concentrations than the ORG group, in which the difference was stable at both day 0 and day 30.

Vitamin C, vitamin D, calcium, zinc, iron, ferritin, and transferrin concentrations remained unchanged across all factors, with no significant effects of time, treatment, or interaction (not significant (NS)) (Figure 2).

### Acute supplementation of organic versus synthetic supplementation

#### Bioavailability of vitamins and minerals

The data reveal a consistent trend of increased nutrient concentrations from rest to 2 h postingestion across most variables for both the ORG and SYN supplements (Table 3). However, all nutrients and minerals showed a nonsignificant interaction effect, indicating acute changes are comparable across treatment groups [*P*(*F*), Table 3] over time.

**TABLE 3:** Mineral and vitamin concentrations for both timepoints in acute trial.

	ORG		*P*(*t*)	SYN		*P*(*t*)	*P*(*F*)
	Rest	2-h		Rest	2-h		
Biotin (nmol/L)	1.22 (0.4)	1.54 (0.5)	0.09	1.69 (1.0)	2.07 (1.2)	0.08	0.88 (0.02)
Folate (μg/L)	4.74 (3.3)	6.06 (3.6)	<0.01	6.61 (3.9)	6.83 (2.8)	0.77	0.55 (0.35)
Vitamin B12 (pmol/L)	76.56 (37.9)	79.95 (38.1)	<0.01	68.36 (25.1)	70.00 (25.3)	0.02	0.80 (0.06)
Vitamin C (ug/mL)	65.94 (24.5)	57.60 (22.5)	0.35	39.06 (17.1)	39.27 (12.6)	0.95	0.36 (0.84)
Vitamin D (nmol/L)	53.83 (19.0)	57.39 (21.7)	<0.01	61.39 (17.4)	64.00 (18.7)	0.03	0.92 (0.01)
Calcium (mmol/L)	2.30 (0.1)	2.45 (0.1)	<0.01	2.41 (0.1)	2.51 (0.2)	0.01	0.44 (0.60)
Zinc (μmol/L)	5.39 (1.7)	6.56 (2.0)	0.03	8.17 (1.3)	9.06 (1.4)	0.04	0.71 (0.13)
Iron (μmol/L)	18.22 (4.8)	22.0 (6.1)	<0.01	17.78 (6.33)	21.44 (6.15)	0.01	0.89 (0.02)
Ferritin (ng/L)	88.50 (61.7)	94.94 (66.58)	0.01	90.17 (58.42)	98.06 (54.48)	0.04	0.96 (0.00)
Transferrin (mg/dL)	277.11 (18.9)	289.83 (23.58)	<0.01	260.94 (36.96)	270.67 (35.36)	0.18	0.72 (0.12)

Mean and SD shown in brackets. *P*(*F*) indicates level of significance for interaction effect for 2-way measures ANOVA, *F* ratio shown in brackets. *P*(*t*) indicates level of significance across time for paired *t*-test.

Abbreviations: ANOVA, analysis of variance; ORG, organically sourced; SYN, synthetically produced.

A significant treatment effect was present for calcium (*P* < 0.001, *F* = 17.90), transferrin (*P* < 0.01, *F* = 7.00), and biotin (*P* < 0.05, F = 6.67), indicating that the SYN supplement group had a consistently slightly higher mean concentration than the ORG group across time points. Conversely, vitamin C had a main treatment effect (*P* < 0.001, *F* = 23.6) indicating that the SYN supplement group had decreased concentrations of vitamin C across the ingestion period. The mean values and SDs for the remaining variables are comparable across ORG and SYN groups across time (NS).

Across timepoints, there was a significant increase in vitamin B12, vitamin D, calcium, zinc, iron, and ferritin. All showed an increase from rest to 2-h ingestion for both supplement types (Table 3). Within the ORG, there is a significant increase in circulatory folate and transferrin concentrations over the 2-h ingestion (*P* < 0.01, *P* < 0.01, respectively); however, this was not present within the SYN group (*P* = 0.77, *P* = 0.18, respectively).

No significant changes in circulatory biotin concentrations or vitamin C concentrations were reported across time in either ORG (*P* = 0.09, *P* = 0.35, respectively) or SYN (*P* = 0.08, *P* = 0.95, respectively).

## Discussion

The present study investigated both acute (2-h) and longer-term (30-d) physiological responses to ORG and SYN MVM supplementation in healthy individuals. A central finding across both intervention phases was the consistent absence of a condition × time interaction effect for the majority of nutrients assessed. This indicates that organic and synthetic formulations elicited statistically indistinguishable trajectories in circulating micronutrient concentrations, thereby supporting the functional equivalence of the 2 supplement types.

This comparability is especially noteworthy in the context of growing consumer demand for “natural” or “clean-label,” and ORG supplements [6]. Global organic food and health-product markets continue to expand, driven by public perceptions that organic products are safer, more health-promoting, and better aligned with environmental and ethical values. Yet to date, limited human data exist comparing the bioavailability or metabolic responses to organically derived compared with synthetically prepared vitamins and minerals, highlighting a significant gap that has contributed to hesitancy within the food, supplement, and functional-food industries [14,15].

Across both study phases, ORG- and SYN-sourced MVM supplements produced similar improvements in circulating micronutrient concentrations across several assessed analytes, consistent with meta-analytic and mechanistic work showing that many vitamins and minerals, whether natural or synthetic, achieve equivalent systemic availability when provided in chemically identical forms [16,17]. The lack of interaction effects in our analyses underscores that the pattern of change and not merely the absolute values was comparable between groups.

Although the present study demonstrates broadly comparable circulating responses to ORG and SYN MVM formulations, the 2 supplements differed substantially in their chemical forms and biological matrices (Table 1). The ORG formulation predominantly provided nutrients in food-derived, chelated, or biologically active forms (e.g., methylcobalamin and adenosylcobalamin, tetrahydrofolate, selenomethionine, or organic acid mineral complexes), whereas the SYN formulation relied largely on inorganic salts or synthetic isolates (e.g., cyanocobalamin, folic acid, sodium selenite, or ferrous sulfate). These compositional differences provide an important framework for interpreting the observed equivalence in circulating micronutrient responses.

Vitamin B12 in the ORG formulation was supplied as a mixture of biologically active cobalamins, whereas the SYN formulation contained cyanocobalamin. Previous research suggests that methylcobalamin and adenosylcobalamin may offer advantages in tissue retention and intracellular utilization, whereas cyanocobalamin requires enzymatic conversion before biological activity [18]. Nevertheless, both forms are efficiently absorbed in healthy individuals with intact intrinsic factor function, and conversion pathways are generally sufficient to maintain circulating status. The consistently higher B12 concentrations observed in the SYN group, irrespective of time point, may therefore reflect differences in baseline status or interindividual variability rather than differential bioavailability.

Folate was provided primarily as tetrahydrofolate (and its derivatives, including L-Methylfolate (5-MTHF)) in the ORG formulation and as folic acid in the SYN formulation. Natural folate derivatives are reported to exhibit distinct absorption and metabolic pathways compared with synthetic folic acid, due to the influence of food-matrix components (e.g., polyglutamate structure, and phytochemicals) in stabilizing folate and supporting uptake [[19], [20], [21], [22]]. However, the current study’s findings demonstrate equivalence of organic and synthetic in terms of circulatory availability.

For most nutrients, including vitamin C, vitamin D, calcium, zinc, iron, ferritin, and transferrin, homeostatic regulation likely contributed to the absence of detectable between-group differences. As highlighted in earlier work, circulating concentrations often remain stable unless dietary intake substantially exceeds physiological requirements, or a baseline deficiency exists [1]. Many minerals and vitamins are regulated by tight homeostatic mechanisms, such that changes in dietary intake, especially over short periods, may not translate to detectable differences in circulating concentrations. However, there are published and disputed alterations in bioavailability for some key markers within the literature.

Several minerals in the ORG formulation were provided in organic acid–chelated or proteinate forms, including iron, zinc, chromium, and selenium, whereas the SYN formulation predominantly used inorganic salts. Chelated mineral forms are often reported to exhibit improved solubility and reduced interaction with dietary inhibitors, potentially enhancing absorption, particularly under conditions of deficiency [23,24]. However, evidence suggests that such advantages are attenuated in individuals with adequate baseline mineral status [23].

Calcium, biotin, and iodine were supplied in chemically comparable forms across formulations, primarily as carbonate, D-biotin, and iodide, respectively. Because these species are widely used in supplementation and exhibit well-characterized absorption kinetics, substantial differences in bioavailability were not anticipated [[25], [26], [27]]. The absence of treatment effects for these nutrients therefore aligns with established physiological expectations and supports the validity of the observed equivalence.

Synthetic vitamin C is often reported as being inferior to naturally sourced vitamin C supplementation, with reports highlighting that it exhibits lower antioxidant capacity and shorter plasma *t*_1/2_ compared with plant-derived vitamin C [28]. However, many of these studies have shown transient and small differences, with limited physiological impact [29,30]. In addition to vitamin C, considerable attention has been given to the bioavailability of vitamin E. Naturally occurring vitamin E (RRR-*α*-tocopherol) is reported to exhibit approximately twice the biological activity of the synthetic racemic mixture (all-rac-*α*-tocopherol) when administered competitively, due to stereochemical differences affecting tissue retention [31]. However, many studies have contested the accuracy of such findings because of factors such as the nature of administration and highlighted equivalent bioavailability when administered in a noncompetitive manner [32].

Given the accelerating shift toward organic health products, the demonstration that organic MVM supplements deliver biochemical outcomes equivalent to synthetic formulations may have significant public health and commercial implications. Consumers often assume that organic micronutrient sources possess inferior efficacy, despite limited supporting evidence [6,8,9]. Our findings provide support that ORG MVMs, when appropriately formulated, can match synthetic preparations in short-term availability, enabling credible positioning of such products within the competitive supplement market; however, future work is needed to assess measures of functionality. Evaluating longer intervention periods, incorporating detailed dietary monitoring, and including functional biomarkers, such as homocysteine (folate/B12), methylmalonic acid (B12), or oxidative status (vitamin C/E), to determine whether subtle differences in chemical form translate to divergent physiological effects. Such mechanistic outcomes will help establish whether nutrient-specific disparities observed here carry clinical relevance over the long term.

Organically derived micronutrients typically exhibit lower nutrient density and potency than synthetic isolates, and so could have limitations in necessitating larger serving amounts and their use in certain delivery formats. Yet, advances in extraction, standardization, and formulation technologies have increasingly enabled their incorporation into commercially viable products. Furthermore, although ORG ingredients may carry higher production and sourcing costs, growing consumer demand for clean-label and sustainably produced supplements may offset modest price premiums and formulation constraints, supporting their adoption in appropriately designed delivery systems [13,15]. Collectively, these findings suggest that ORG MVMs, when carefully formulated, can represent a feasible alternative to synthetic preparations, although further research incorporating long-term functional and technoeconomic outcomes is warranted.

In conclusion, this study demonstrates that organic and synthetically derived MVM supplements are broadly comparable in their short-term availability, with no evidence of differential time-dependent responses. Where differences were observed, they aligned with established nutrient-specific biochemical behavior rather than reflecting the intrinsic superiority of either source. These findings provide timely, practical insight for nutrition science, public health recommendations, and the expanding organic-supplement industry, affirming that organically derived MVMs can serve as equally effective with respect to absorption, and an alternative to synthetic formulations, particularly in light of strong consumer preference for organic health products.

### Study limitations

The primary limitation within this study is a lack of stringent control on dietary influence. However, the study aimed to establish the pilot exploration of organically versus synthetically formulated MVM supplementation within a real-world setting, in which the data provide pivotal insight. A secondary limitation was with baseline sufficiency among participants, which may also have limited the capacity for measurable increases. Future work would aim to include a comprehensive and powered randomized controlled trial in individuals with MVM deficiencies, which incorporates dietary control and analysis across the supplementation period.

## Author contributions

The authors’ responsibilities were as follows – RMB, JPT: designed the research; RC, RMB, JPT: performed material preparation, data collection, and analysis; RC: wrote the first draft of the manuscript; RC, ES, JPT, RMB: reviewed and commented on previous versions of the manuscript; and all authors: read and approved the final manuscript.

## Declaration of generative AI and AI-assisted technologies in the writing process

The author(s) declare that no generative AI or AI-assisted technologies were used in the writing of this manuscript.

## Data availability

The datasets used and/or analyzed during the current study are available from the corresponding author on reasonable request.

## Funding

This research received funding from Innovate UK for the completion of the trial.

## Conflict of interest

ES is an employee of BIOVIT, Ltd., who supplied supplementation within the study.
